# Extracellular Vesicles and Their Potential Use in Monitoring Cancer Progression and Therapy: The Contribution of Proteomics

**DOI:** 10.1155/2019/1639854

**Published:** 2019-06-09

**Authors:** Maria Concetta Cufaro, Damiana Pieragostino, Paola Lanuti, Claudia Rossi, Ilaria Cicalini, Luca Federici, Vincenzo De Laurenzi, Piero Del Boccio

**Affiliations:** ^1^Department of Pharmacy, University “G. d'Annunzio” of Chieti-Pescara, Chieti, Italy; ^2^Analytical Biochemistry and Proteomics Laboratory, Centre on Aging Sciences and Translational Medicine (Ce.S.I-MeT), University “G. d'Annunzio” of Chieti-Pescara, Chieti, Italy; ^3^Department of Medical, Oral and Biotechnological Sciences, University “G. d'Annunzio” of Chieti-Pescara, Chieti, Italy; ^4^Department of Medicine and Aging Sciences, University “G. d'Annunzio” of Chieti-Pescara, Chieti, Italy; ^5^Cellular and Molecular Biochemistry Laboratory, Centre on Aging Sciences and Translational Medicine (Ce.S.I-MeT), University “G. d'Annunzio” of Chieti-Pescara, Chieti, Italy

## Abstract

Extracellular Vesicles (EVs) are small membrane-enclosed particles released by cells and able to vehiculate information between them. The term EVs categorizes many and different vesicles based on their biogenesis and release pathway, such as exosomes (Exo), ectosomes, or shedding microvesicles (SMVs), apoptotic blebs (ABs), and other EVs subsets, generating a heterogeneous group of components able to redistribute their cargo into the entire organism. Moreover EVs are becoming increasingly important in monitoring cancer progression and therapy, since they are able to carry specific disease biomarkers such as Glypican-1, colon cancer-associated transcript 2, CD63, CD24, and many others. The importance of their biological role together with their heterogeneity prompted researchers to adopt and standardize purification methods able to isolate EVs for characterizing their cargo. In this way, mass spectrometry (MS)-based proteomics approaches are emerging as promising tool for the identification and quantification of EVs protein cargoes, but this technique resulted to be deeply influenced by the low quality of the isolation techniques. This review presents the state-of-the-art of EVs isolation, purification, and characterization for omics studies, with a particular focus to their potential use in monitoring cancer progression and therapy.

## 1. Introduction

Both eukaryotic and prokaryotic cells release spherical particles enclosed by a phospholipid bilayer into the extracellular space. It is becoming increasingly clear that these Extracellular Vesicles (EVs) have specialized functions and are involved in many cellular processes such as intercellular communication, cellular homeostasis, coagulation, and waste management [1]. Therefore, their involvement in different pathophysiological processes has been investigated in several excellent works highlighting that EVs can be potentially used for diagnosis, prognosis, and therapy as putative biomarkers for health and disease in modern preventive and precision medicine.

The discovery of EVs can be traced back to initial studies concerning blood coagulation. Peter Wolf, one of the key contributors to the definition of the coagulation cascade, was the first to define some subcellular coagulant materials as “platelet dust” [2]. In 1967, he isolated and characterized this material from blood samples through a series of ultracentrifugations, separation, and coagulant experiments. He concluded that “platelet dust”, identified by electron microscopy, possessed coagulant properties, like Platelet Factor 3 (PF3) [3]. Afterwards the “platelet dust” has been called generically “microparticles” (MPs) or “microvesicles” (MVs).

The term EVs categorizes many and different vesicles based on their biogenesis and release pathway, such as exosomes (Exo), ectosomes, or shedding MVs, apoptotic blebs (ABs), and other EVs subsets [4]. Therefore, in the first part of this review we will clarify both the EVs nomenclature, as it was suggested by the International Society for Extracellular Vesicles (ISEV, https://www.isev.org), and all the standardized known methods to isolate them. As a matter of fact, the whole context on EVs data was discussed by ISEV, a group of scientists with a long-term expertise in the field of EVs biology. A set of criteria on their characterization has been proposed that all investigators should adopt in their scientific works [5].

The composition of EVs is not casual, but related to the molecular fingerprint of the cell that originates them and to disease-type. As a matter of fact, EVs are able to transmit specific signals to recipient cells through the proteins, lipids, nucleic acids, and sugars they contain, so that they are thought to represent specific molecular mediators of extracellular communication [6–8]. Notably, it is now evident that tumor cells release different subtypes of EVs, including cancer-derived EVs termed “large oncosomes” (LOs) that may present new perspectives for tumor profiling [9].

**Figure 1:**
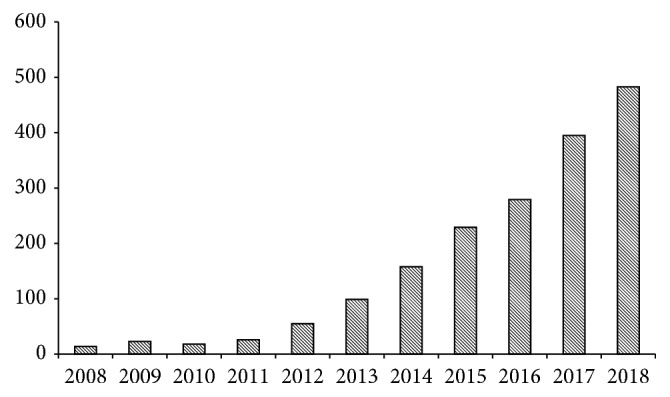
Number of papers published in the last decade in cancer EVs research.

The emerging role of EVs in cancer understanding is demonstrated by the exponentially increase of published papers in the last decade on this topic. Actually, by using “cancer and EVs” as keywords for a search within the SCOPUS database (https://www.scopus.com), the increase of publications in this area is evident as reported in Figure 1, proving the mounting interest of researchers in the comprehension of this new phenomenon.

Here we provide an overview about the biogenesis and composition of main EVs, along with their cancer-specific and general functions.

While in the past EVs were typically isolated from cultured cell lines, nowadays they can be isolated from most body fluids, including blood, urine, saliva, amniotic fluid, semen, and tears [10–13]. The number of EVs in biological fluids seems to be correlated with the active phase of many disorders and diseases; thus MVs and Exo are currently under investigation for their clinical use as possible biomarkers or as adjuvant therapy [1, 14, 15]. Their potential use, related to monitoring disease progression in real time, is strengthened by the possibility of analyzing them in biological fluids through minimally invasive extraction techniques, such as blood, saliva, urine, or tears [16].

Finally, in this review, we want to provide a detailed view on the EVs and their world around from the isolation and purification methods to the characterization techniques. Since analysis of vesicular biocargo requires very pure EVs preparations, we will explain how it is possible to better characterize the protein cargo of isolated EVs by focusing on the advantages and disadvantages of the various isolation techniques, especially with the use of mass spectrometry (MS)-based proteomics approaches.

## 2. The Various World of Extracellular Vesicles

**Figure 2:**
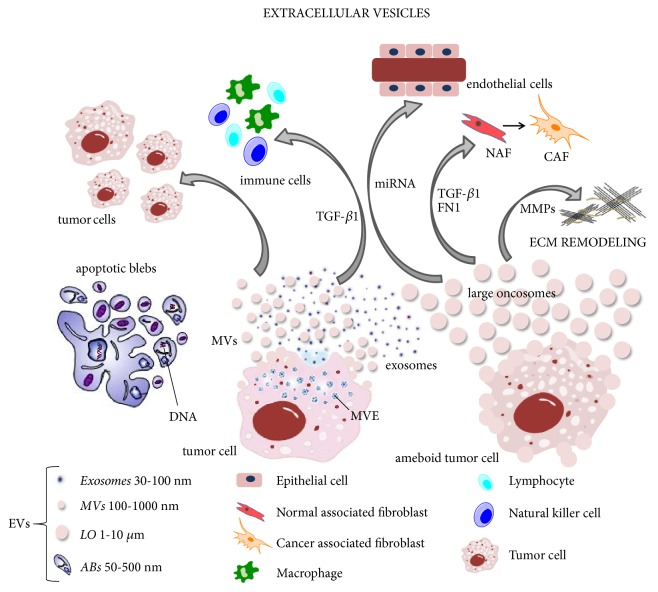
*Large oncosomes: the new players in intercellular communication for tumor progression and metastasis*. Tumor cells communicate each other and with neighboring normal cells in their microenvironment by sending out biological signals enclosed in EVs. Large Oncosomes are membrane vesicles released from “ameboid” tumor cells that are able to facilitate migration of tumor cells and promoting metastasis. The figure shows how specific tumor-cell EVs are involved in tumor progression by targeting fibroblasts and endothelial and immune cells or by altering the structure and composition of ECM. EVs, Extracellular Vesicles; MVs, microvesicles; LOs, large oncosomes; ABs, apoptotic blebs; MVEs, multivesicular endosomes; ECM, extracellular matrix; TGF-*β*, transforming growth factor beta; FN1, fibronectin-1.

In the past decade, EVs have been recognized as important molecular messengers in many pathophysiological processes. However, the extracellular microenvironment contains a mixed population of EVs that have been categorized by their different structure and biochemical properties with confusing terminologies [17]. Therefore, it is important to provide a correct and stringent classification of EVs in order to avoid confusion and cross-contamination in isolating specific vesicular subsets. Firstly, towards a standardizing nomenclature, it is useful to distinguish EVs according to their chemical and physical characteristics, such as size, density, lipid composition, main protein markers, morphology, molecular cargoes, subcellular origin, and release mechanism [1, 18]. For this reason, the ISEV suggested to classify EVs into three different main groups [5, 19]: (1) Exo; (2) MVs, also called shedding vesicles, shedding microvesicles (SMVs) [17] or microparticles (MPs) [1]; and (3) apoptotic bodies or apoptotic blebs (ABs), also called apoptotic vesicles (AVs) [20]. The term exosome was initially used for membrane vesicles ranging from 40 to 100 nm, but their origin was still unclear. Nowadays, it is clear that the formation of EVs is a tightly regulated process [7]. In fact, the modern scientific literature refers to Exo as EVs with endocytic origin released from multivesicular endosomes (MVEs), while MVs are formed by the blebbing of the plasma membrane and subsequent fission of membrane blebs [1, 17]. The main characteristics of the different types of EVs are summarized in Table 1. The release mode of the main EVs subpopulations is shown in Figure 2, together with their involvement in cancer progression and metastasis.

### 2.1. Exosomes (Exo)

Exo are cell-derived membrane vesicles that have been found in almost all biological fluids, such as urine, blood, and cerebrospinal fluid, and have been isolated mainly from culture medium of cell cultures [1]. They were firstly reported in 1983 by Johnstone and colleagues as small membrane-enclosed vesicles with transferrin receptor while culturing and maturing reticulocytes [21–23]. Exo show a small diameter (between 40 and 100 nm) [17, 23] and they are usually isolated through ultracentrifugation. Exosome morphology has been described as cup-shaped through fixation, negative staining, and visualization by Transmission Electron Microscopy (TEM) [1]. Regarding their biochemical composition, Exo are surrounded by a phospholipid membrane with high levels of cholesterol, sphingomyelin, and ceramide. Moreover, they are characterized by the presence of proteins involved in membrane transport and fusion, associated to the endosome formation, (such as Rab, GTPases, and Annexins), by components of the Endosomal Sorting Complexes Required for Transporter (ESCRTs) and by tetraspanins, including CD9, CD63, and CD81 [1, 17, 23].

The “classic pathway” of Exo biogenesis involves the formation of intraluminal vesicles (ILVs) within large MVEs [1] or multivesicular bodies (MVBs) [17]. The intracellular MVEs can merge to lysosomes for cargo degradation (i.e., “degradative MVEs”) or with the plasma membrane to secrete ILVs into the extracellular space (the so-called “exocytic MVEs”); these excreted vesicles are then referred to as “Exosomes” [1, 17, 20]. Together with the “classic pathway” of exosome biogenesis, there is also a second route of exosome formation, directly from cellular plasma membrane. The vesicles obtained through this way are indistinguishable from the other Exo formed by the classic endosomal pathway because they have a similar diameter and density and are also enriched in classic exosome markers such as CD63, CD81, and CD9 [1]. In particular, the ILV/Exo formation requires two different steps [20]. The first step involves the organization of the endosome membrane into specialized units enriched of specific membrane proteins called tetraspanins. These particular regions are termed tetraspanin-enriched microdomains (TEMs). The tetraspanins CD9 and CD63 play an important role in Exo formation; therefore, they are usually used as Exo markers for their isolation. The second step is linked to a series of specific complexes called ESCRTs. In particular, there are four multiprotein complexes responsible for ILV formation that are called ESCRT-0, -I, -II, and –III [20, 23]. The knowledge about Exo biogenesis is still fragmentary but ESCRTs appear to be implicated in both exosomal ILV budding and cargo loading. It has been demonstrated that Alix, an ESCRT-accessory molecule, uses some of its protein motifs to interact with the cytoplasmic adapter protein Syntenin [24]. Syntenin, in turn, binds the trans-membrane Heparan Sulfate Proteo-Glycan (HSPG) Syndecan. The Alix/Syntenin/Syndecan complex is involved in the exosomal cargo selection, as well as in ILV formation, allowing the endosomal invagination which leads to the genesis of ILVs that contain the Exo cargoes [25]. Syndecans can interact with molecules implicated in cell adhesion and signaling, therefore recruiting specific cargoes [26]. The Alix/Syntenin/Syndecan complex also produces the physical biogenesis of exosomal ILVs. It has been demonstrated that the overexpression of these factors is associated to a decrease of the ILV formation and to the Exo release [24]. The ESCRT machinery is also involved in the ubiquitylation of some membrane proteins that may actually serve as internalization signal, because it prevents the recycling of ubiquitylated cargoes [27]. In particular, the ESCRT-0, -I, and -II complexes recognize and sequester ubiquitinated membrane proteins [23, 25, 28]. Likewise, next to the ESCRT machinery, there is an ESCRT-independent mechanism for exosome biogenesis and release that depends either on the sphingolipids, ceramides, and its molecular pathway or on TEMs, already described above [1, 23].

### 2.2. Microvesicles (MVs)

Unlike Exo, MVs are originated directly from the plasma membrane and they are often categorized as ectosomes. In order to give a clear nomenclature, Mathivanan et al. [17] distinguish, in their review, the SMVs as large membranous vesicles with a diameter greater than 100 nm that are shed from plasma membrane by its budding/blebbing. Shedding vesicles formation takes place from the budding of small cytoplasmic protrusions followed by their detachment from the cell surface [29]. This process involves a dynamic interplay between phospholipid redistribution and cytoskeletal protein contraction thanks to flippases and floppases that allow the translocation of phospholipids [7, 23, 28]. Then, the release of MVs is efficiently regulated and induced upon activation of cell surface receptors or apoptosis and the subsequent increase of intracellular calcium ions [7, 28, 30]. Van der Pol et al. [1] use the term “microvesicles” or “microparticles” to describe the larger population of membrane vesicles ranging from 100 nm to 1 *μ*m released from plasma membrane during cell stress. The term “microparticles” has also been used for total populations of vesicles isolated by human plasma through ultracentrifugation [1]. In particular, cancer-derived MVs are enriched in ADP-ribosylation factor 6 (ARF6), which promotes MVs shedding from plasma membrane of prostate and breast cancer cell lines [31].

### 2.3. Apoptotic Bodies (ABs)

The term “apoptotic bodies” was coined by Kerr in 1972 [32]. They are membrane vesicles released into the extracellular environment when cells are undergoing apoptosis during the last stages of cell death [1, 17]. The major difference between ABs and other cell-derived vesicles is their size because they are 1-5 *μ*m in diameter [1, 20, 30]. Moreover, they are heterogeneous in shape [1, 17] and present intact organelles, histones and genomic DNA within the vesicles [20]. In general, ABs are released by membrane blebbing that requires phosphorylation of myosin light chain and the activation of caspase-3, one of the key enzymes of apoptosis [1].

### 2.4. Large Oncosomes (LOs)

Large oncosomes (LOs) display a diameter between 1 and 10 *μ*m and are therefore much larger than most EVs types. Similarly to MVs, they express ARF6 and originate directly from plasma membrane budding, but LOs are nonapoptotic membrane blebs that shed from aggressive cancer cells. Their formation and release are enhanced by the loss of the cytoskeletal regulator diaphanous related formin-3 (DIAPH3), which induces a transition from a mesenchymal to a more rapid, invasive and metastatic “amoeboid” phenotype [54, 55]. For this reason, LOs can be associated with tumor progression according to the oncosome-mediated plasticity of amoeboid cells [9, 33]. As a matter of fact, only tumor cells release a quantifiable amount of LOs and this appears to correlate with tumor aggressiveness, whereas their detection in benign cells is negligible. Their shedding is common to several tumor types, including prostate, breast, bladder, lung cancer, and others [31]. They were also identified in the circulation of mice and in the plasma of patients with metastatic prostate cancer, suggesting that these membrane vesicles could be sources of clinical biomarkers [33, 37, 55]. LOs contain significantly more abundant extracellular DNA than small cancer EVs, like Exo, not only in* in vitro* cultured tumor-cell lines but also in plasma, demonstrating that large EVs-derived DNA reflects the genomic make-up of the origin tumor cells [37]. Also mRNA and miRNA have been identified within LOs, suggesting that upon internalization by recipient cells, they can regulate gene expression and enhance the migration of fibroblasts [31, 36].

In particular, their abundant biological content may affect cell metabolism, mRNA processing, cell growth, and motility. These giant vesicles were identified both in breast cancer lines and in tissue sections of human breast cancer showing their probable involvement in tumor progression and metastases because of the overexpression and export of oncogenic protein complexes among tumor cells or between tumor cells and stroma [33, 36].

## 3. Biological Role of Extracellular Vesicles: Focus on Cancer

### 3.1. Role of EVs in Cell-to-Cell Communication

Growing experimental evidences indicate that EVs are underappreciated cellular components and may play key roles in many biological processes especially as mediators of intercellular communication and exchange of signaling components. Indeed, the intercellular communication mediated by Exo (and more in general by all EVs) has gained considerable attention, in light of the importance of understanding the multiple languages of cell-to-cell communication especially in tumor cells and in therapy response [56]. Based on these considerations, EVs have been proposed for emerging clinical applications as biomarkers, direct therapeutic targets, and engineered nanocarriers. These roles are due to fact that during their formation, EVs become enriched in many molecules that are expressed in the cytoplasm and on the membrane of the cells of origin. Cellular interactions mediated by membrane vesicles are pivotal for cell growth and development, for cellular proliferation, differentiation and senescence, and for many pathophysiological processes, such as progression, angiogenesis, invasiveness and metastasis of tumors [1, 17, 29, 34, 57], inflammation and immune modulation [1, 58, 59], neuroprotection and regeneration after injury, and disease progression in central nervous system disorders [60]. These evidences highlighted the possibility that such EVs may suggest such biological functions to specific target cells, actively acting communication between cells. In conclusion, cell-derived membrane vesicles seem to separate and protect their contents from extracellular environment allowing their cargoes to be transported intact and to form a communication network between neighboring cells and distant cells [8, 36]. Furthermore, EVs may contribute to the pathogenesis of various diseases because these vesicles can be equipped with cell type-specific adhesion receptors so that their cargo will be delivered only at dedicated target cells [12]. EVs contain materials related to their cells of origin, therefore isolating EVs and analyzing their biological content from body fluids may be a precious source of information.

### 3.2. Role of EVs in the Immune System Modulation

EVs are widely involved in the transfer of infectious agents [58] since they transfer viruses' receptors, that are essential for infection and survival in the infected cells, and also because of their action as potential decoys to elude the immune system [1]. The role of EVs in the immune system modulation has been particularly studied in cancer, since tumor exosomal compartment is able to evade immune system by cell killing at a distance [1]. Fas ligand (FasL) is present in EVs of melanoma cells [61], prostate cancer cells [62] and in epithelial ovarian cancer cells. Moreover, they are all capable of inducing T-cell apoptosis [27]. Indeed, Kim et al. [63] also demonstrated that FasL-exposing EVs from sera of patients with oral squamous cell carcinoma induce T-cell apoptosis.

### 3.3. EVs in Tumor Progression and Metastasis

In 1978, Friend and colleagues were the first to show the ability of tumor cells of shedding membrane vesicles in Hodgkin's disease. Moreover, such vesicles were described as “rare pleomorphic particles ranging from 400 to 1200 Å” [64]. Only twenty years later, it was proven that these “particles”, called EVs, are not artifacts, but they are involved in specific phases of tumorigenesis, tumor development, growth, survival, and progression [65]. In 2008, Al-Nedawi et al. [66] demonstrated that the oncogenic form of the epidermal grow factor (EGFRvIII), which is specific to human glioblastoma, is released from brain tumor cells as cargo of EVs ranging between 100 and 400 nm in diameter.

During primary tumor formation, cancer cells are able to communicate with each other and with neighboring normal cells in their microenvironment by sending out signals in the form of cytokines, signaling proteins and EVs [67]. For this reason, EVs are object of active investigation by scientists for a better understanding of their biological role* in vivo*. Accordingly, in this review we summarize the horizontal communication between cells for tumor progression and metastasis, because oncogene transcripts and miRNAs can be transported through EVs and then translated into proteins in the recipient cells. While some of the most common mechanisms of interaction between tumor-derived MVs and Exo with target cells have been described in many studies [1, 34, 57], the cross talk between LOs and the microenvironment is still unknown. In general, tumor-cell specific EVs are emerging as potential source of cancer biomarkers because they transport bioactive molecules able to alter the homeostasis of the tumor microenvironment by targeting fibroblasts, endothelial and immune cells [9, 31, 67] (Figure 2). First of all, it is important to underline that many studies demonstrated that tumor EVs can functionally modify fibroblasts by giving them a cancer phenotype. In fact, these cells are reprogrammed to cancer-associated fibroblasts (CAFs) because tumor EVs transport active molecules, such as transforming growth factor beta (TGF-*β*) [33, 36, 68], fibronectin-1 (FN1), and tissue transglutaminase (tTG) [69] and may promote tumor progression and cancer cell invasion. At the same time, EVs secreted by the stroma in the tumor microenvironment can also promote tumor motility, invasion, and dissemination of cancer cells [36].

The metalloproteases (MMPs) are a family of proteins implicated in extracellular matrix remodeling and in cancer cell protease-dependent migration and invasion. They are both activated inside and on the EVs membrane [70]. Di Vizio et al. [55] showed that prostate cancer cell-derived oncosomes contain bioactive MMP9 and MMP2, suggesting that LOs could have a key role in facilitating migration of tumor cells and promoting metastasis [56].

### 3.4. EVs in Monitoring Cancer Therapy

The biological role of EVs allowed the study of EVs as potential biomarkers for monitoring cancer progression and therapy. Actually, EVs represent relevant tumor circulome constituents with promising potential at each stage of cancer management [71]. For example, it has been recently demonstrated that EVs may be useful as a predictive biomarkers for Anti-PD-1 (programmed death-1) therapy outcome. Moreover, cancer-derived EVs transfer functional programmed death-ligand 1 (PD-L1) and may be both regulators and biomarkers of therapy resistance [72].

Notably, recent findings [73] support the idea that tumor EVs may also induce an immunosuppressive microenvironment since Galectin-1 (Gal-1), which is present in tumor EVs, reduce T-cell infiltration in the tumor microenvironment [74].

On the other hand, EVs have been also proposed as new forms of treatment for different diseases, given that EVs are natural systems able to deliver biological messages to target cells. The goal of the research in this field is to substitute the biological messages into therapeutic molecules. To achieve this goal, different aspects should be taken into account, such as the improvement of biotechnology techniques, the choice of EV cellular source, loading, isolation methods, and engineering approaches for drug targeting [75].

Interestingly, it has been demonstrated that the cells incubated with chemotherapeutic molecules are able to package these compounds into EVs. In this context, it has been demonstrated that the use of human red blood cells to produce EVs for RNA therapies is particularly suitable. In fact, red blood cells belonging to O-group are largely available and, given that they do not contain DNA, there is no risk for horizontal gene transfer. It has been also demonstrated that a large amount of red blood cell-derived EVs can be isolated and electroporated with antisense oligonucleotides directed to miR-125b-2, or Cas9 mRNA and gRNA targeting the miR125b-2 human locus [76]. All those engineered EVs which resulted are able to inhibit both* in vitro* and* in viv*o leukemia and breast cancer cell proliferation.

### 3.5. EVs as Cancer Biomarkers

It has been suggested that total circulating EVs are able to identify cancer disease at very early stages. For example, Exo containing Glypican-1 (GPC1) is very sensitive and specific biomarkers of pancreatic cancer in blood of patients [77].

Intriguingly, colon cancer-associated transcript 2 (CCAT2) expression was found upregulated in Colorectal Cancer (CRC) Exo [78]. Moreover carcinoembryonic antigen (CEA) and epithelial cell adhesion molecule (EpCAM) are both highly expressed on the surface of CRC-derived Exo [79, 80]. The ability of Exo in miRNAs deliver is widely demonstrated in many studies [81]. In particular, serum miR-21 is highly expressed in CRC-derived Exo and it can be a possible marker for early stage diagnosis [82] even if not highly specific for this kind of cancer [83].

Furthermore, new evidences show that Exo are important in gastric cancer development and progression [84]. The role of Exo in Gastric Cancer was already reported by Qu et al. [85], in 2009, describing that cell-derived Exo promoted cell proliferation through PI3K/Akt and MAPK/ERK pathways. Recently, CD63 has been considered as a prognostic marker for patients with gastric cancer and CD63+ Exo might be associated with the interaction between stromal cells and cancer cells [86].

Even breast cancer (BC) Exo have been studied as potential disease biomarkers since high levels of exosomal CD24 were shown in serum from patients. However, CD24 has been implicated in numerous cancer types so it may serve as a general cancer marker and not as a specific biomarker for BC [87]. The ability of EVs in carrying BC biomarkers was widely studied [88] and many BC circulating biomarkers have been reported. For example, fibronectin enriched EVs were studied as putative biomarkers for early diagnosis of BC [89]. Along the same line, developmental endothelial locus-1 (Del-1) on circulating EVs was identified as a promising marker to improve identification of patients with early stage BC, even in discriminating BC from benign breast tumors [90].

These data represent only a small part of the literature on EVs for cancer biomarker discovery. Indeed, hundreds of papers have been written on this topic and we anticipate that these numbers will increase in the next years, since EVs represent a circulating magnifying glass of tumor complexity and carry precious information on tumor development which is easily and noninvasively extractable from biological fluids.

## 4. Methods to Isolate Extracellular Vesicles

Since Peter Wolf's original descriptions, studies on EVs have increased exponentially because of the major interest in the biological role of these membranous vesicles. Many questions have arisen about the analytical techniques used to isolate and separate EVs because the methods used for their isolation can greatly influence the analysis of their composition. Therefore one main goal in the field is the choice of the correct isolation technique that would avoid or minimize cross-contamination.

### 4.1. Choice of Body Fluids for EVs Isolation

It is important to take into consideration that during EVs isolation possible contaminants may appear, especially protein-contaminants, such as filamentous proteins and protein aggregates that may coisolate during the separation steps [19]. Many studies on Exo, and generically on EVs, have been performed using supernatants of cultured cells, since the origin of EVs is cell-defined and it can be determined by immunophenotyping experiments. On the contrary, isolation of EVs from plasma or other body fluids is more difficult for many reasons [5, 91]. First of all, biological fluid-derived EVs are produced by a vast array of cells in tissues and in varying proportions; secondly, isolated EVs could be “coated” with proteins, glycoproteins or glycolipids likely to cause aggregation, fusion and cosedimentation of the same vesicles [30, 91]; thirdly, the presence in the body fluids of abundant and typical proteins can influence the recovery and purity of isolated EVs, thus impeding an accurate and efficient analysis [10, 19]. For example, plasma is widely used as a specimen, but it contains abundant albumin, immunoglobulins and lipoproteins that can negatively affect proteomics analysis if not properly separated from EVs. For this reason, the information obtained from enriched EVs could be also influenced by the environment, especially by the type of fluid in which they can be isolated [92]. As EVs are gaining prominence in the “liquid biopsies” field [92], in this review we discuss the various techniques used to separate them in the main complex body fluids. Certainly, the most common biofluids for liquid biopsy may be plasma and serum, since they are relatively easy to collect. Serum and plasma may be particularly advantageous in cancers with precarious location of the lesions as, for example, non-small-cell lung cancer and brain tumors [92]. Another advantage of serum and plasma EVs may be the study of novel circulating biomarkers, supplementing prostate specific antigen (PSA) [53], in order to improve biomarkers' sensitivity and specificity in the early disease stages [92]. On the other hand, urinary EVs are easy to obtain since urine is a cleaner biofluid, leading to simpler purification procedures. They may be used not only for the enrichment of urogenital system diseases biomarkers [92], but also for the systemic diseases because their protein composition reflects the status of the circulatory system [93]. Furthermore, EVs isolated from cerebrospinal fluid (CSF) may be considered as new diagnostic tools in central nervous system (CNS) disorders representing a “brain fluid biopsy” [60, 94]. Even tears and saliva may be useful source of biomarkers thanks to the smaller protein dynamic range of these biofluids in respect to serum and plasma [92]. Actually tears and salivary EVs represent attractive medium for diagnosis and monitoring diseases using proteomics analysis [11].

### 4.2. Choice for EVs Isolation Technique

In the last decades, various methods have been developed to isolate EVs both from cell cultures and biological fluids. These methods differ in yield, purity and size distribution of isolated EVs [10, 95]. It has been observed that the method used to isolate and purify EVs can influence their number, integrity and impact on their subsequent biodistribution* in vivo *[91]. Rosa-Fernandes* et al*. [6], recently reviewed the major EVs isolation methods, especially as regards their advantages and disadvantages in the light of subsequent MS-based proteomics analysis. Actually, no general consensus was found regarding protocols for EVs isolation. As a consequence, optimization of the separation protocol for clinical purposes is absolutely necessary. Many of the isolation methods described in Table 2 can be combined in appropriate workflows.

To date, there is no EVs isolation technique that allows the recovery of a completely pure EVs subpopulation [6]. However, the recent scientific literature aims at standardizing and refining the isolation methods to separate specific vesicular subclasses in order to maximize purity and recovery.

In the following sections we discuss the merits and demerits of various techniques used to isolate EVs, especially regarding their compatibility with following proteomics strategies based on liquid chromatography-mass spectrometry analysis (LC-MS).

#### 4.2.1. Ultracentrifugation

To date, ultracentrifugation (UC) is the classical and most commonly used method to isolate EVs [17, 19, 38]. It uses a strong centrifugal force to separate EVs forming a pellet at the bottom in an ultracentrifuge tube. UC can be categorized based on the principles of separation: differential centrifugation (DC) and density gradient centrifugation. DC has many disadvantages since it is slow and laborious; the presence of contaminants in the vesicular pellet requires additional centrifugation steps that can induce aggregation of vesicles and the coisolation of protein aggregates that decrease the amount of isolated EVs and the purity of the sample due to high rotation speed [6, 10]. In conclusion, with DC it is really difficult to get clean EVs because of the presence of clusters of other non-EV components [19]. In fact, while some groups suggest that UC causes vesicles aggregation and fusion resulting in false diameter readings, some others maintain that not performing the UC step leads to contaminated samples [2, 10]. According to Linares and colleagues the interpretation of data obtained after high-speed centrifugation must be taken with caution because of the possibility of EVs aggregates formation that might affect either the purity and concentration of EVs or their size and biochemical composition [39].

However, density gradient centrifugation is less affected than differential centrifugation by protein contamination because proteins are accumulated into different density layers as compared to the EVs, based on size and mass density [6, 10, 19, 40, 41]. Iodixanol and sucrose are the most commonly used density medium [6, 41].

#### 4.2.2. Ultrafiltration

One of the popular size-based Exo (and generally of EVs) isolation techniques is ultrafiltration which captures EVs on membranes allowing small particles, like proteins, to pass through them [10, 40]. In particular, sequential filtration is applied especially to isolate Exo from cell culture supernatants [40]. It is commonly used as a first step to concentrate vesicular population from a large volume of starting material into a more manageable volume which can be used for others purification methods, such as UC and size-exclusion chromatography (SEC) [13, 40, 42]. However, generally, isolation based on size cannot discriminate EVs from cellular debris, small vesicles, protein aggregates, or subpopulations of similarly sized EVs [10]. Membrane filtration leads to sample loss due to unspecific membrane adsorption, but it can be used to clean up the sample before LC-MS analysis [6].

#### 4.2.3. Immunoaffinity Capture

Immunoaffinity capture exploits interaction between antibodies and surface vesicular proteins to isolate EVs. Tetraspanin-specific antibodies are commonly used in immunoaffinity capture of defined Exo markers (CD9+, CD81+, and CD63+). Indeed, while size-based separation cannot distinguish among subpopulations of EVs, immunoaffinity is able to distinguish, for example, the CD81+ subpopulation of EVs from the CD63+ one. Antibodies specific to surface proteins of EVs are linked to chemically modified or protein-coated beads [10, 20, 40]. Immunoaffinity is not suitable for sorting by size, but it is particularly useful to characterize the vesicular phenotype by investigating their specific surface proteins, which are related to the cell of origin. By combining DC, filtration and immunoaffinity capture, Exo have been isolated from both cell cultures and body fluids [17, 96]. For this reason, a commercial exosome isolation kit has been developed based on the concept of magneto-immunocapture [40]. As to the disadvantages, antibodies are very expensive and structural damage may occur in the displacing the EVs from the beads [10, 16].

#### 4.2.4. Precipitation

Currently, several exosome (more in general EVs) precipitation kits are commercially available, such as Exoquick™ (System Biosciences) and Total Exosome Isolation kit (Thermo Fisher Scientific) [97, 98]. Some of them are compatible with body fluids including serum, urine, plasma, cerebrospinal fluid, and culture medium. Exo are settled out of biological fluids by altering their solubility with water-excluding polymers such as polyethylene glycol (PEG) and dextran. This method is defined aqueous two phase system (ATPS): after centrifugation EVs move into a dextran phase that has favorable surface properties to allow their purification. ATPS is a quick and easy method, showing higher yield of EVs than UC and nanomembrane concentrators [10, 40, 99]. Ymir Genomics developed a novel, proprietary and rapid precipitation method that does not require PEG. It is used to isolate EVs and extracellular RNA from urine [99]. However, in order to remove the contaminating proteins and to clean-up samples, a size-exclusion chromatography step is required. An example of polymer-based precipitation with size-based purification (PBP+SP) is Exo-spin™, Exosome Purification kit (Cell Guidance Systems) that allows obtaining EVs preparations with decreased protein contamination [100]. Anyway, EVs isolated with precipitation methods contain excess of salts and polymer incompatible with direct MS analysis, requiring in-gel digestion purification or membrane filtration to clean up the sample before analysis [6, 16].

PRotein Organic Solvent PRecipitation (PROSPR) is a novel exosome isolation method that uses organic solvents (cold acetone, chloroform, trichloroacetic acid) to precipitate proteins from biological fluids with compatible solvents for MS. Moreover, it is important to underline that the precipitation methods result in higher protein amount than the UC method [6, 10, 101, 102]. All these steps require sample manipulations that are very difficult to control in a quantitative proteomics approach.

#### 4.2.5. Size-Exclusion Chromatography

Another size-based separation technique applied to EVs isolation is size-exclusion chromatography (SEC), which uses a column packed with a porous stationary phase. Small particles enter the pore and move slowly, whereas larger particles pass through the column rapidly, leading to elution in order of decreasing size [10, 16, 40]. Because of the high sample volume, SEC is followed by ultrafiltration to separate the EVs from interfering molecules contained in complex body fluids, such as saliva, tears [11] and CSF [94]. Rood et al. [103] showed that UC followed by SEC allows enriching urinary Exo in comparison to the yields obtained by UC or ultrafiltration alone. SEC separates the vesicles from soluble proteins and small molecules, so that it gives higher pure EVs than PROSPR or polymer-based precipitation methods in which precipitating agents could alter EVs structure and composition [6].

#### 4.2.6. Microfluidics Techniques

With microfluidics-based devices it is possible to separate Exo rapidly and efficiently, exploiting new innovative sorting mechanisms such as acoustic, electrophoretic and electromagnetic manipulations [40]. Microfluidics techniques are combined with immunoaffinity methods to detect EVs by sieving or trapping them in porous microsized channels [10]. For example, a commercial product is now available: ExoChip [104] is an immunochip funzionalized with antibodies against CD63, a common Exo marker. It is able to isolate Exo with intact RNA for analysis of exosomal miRNA profiling [10, 40, 104]. An example of microfluidic technique uses filter membranes with porous polymer monoliths (PPM) that can isolate EVs from mouse blood. Filtration can be classified as pressure-driven or electrophoresis-driven. The latter exploits the negative charge of phospholipid membranes of EVs to move them across filter membranes in order to be evaluated and then collected. Proteins and other molecules that have different charges are separated from EVs. Electrophoresis-driven filtration is not blocked by any molecule and especially by gas bubbles [10]. Wang* et al*. [105] fabricated a porous silicon nanowire-on-micropillar structure made up of ciliated micropillars that purifies EVs with minimal contamination. Ciliated micropillars on the wall filter lipid vesicles like Exo, whereas the larger vesicles, cellular debris, and small molecules like proteins are passed in a continuous flow [10, 40]. EVs purification using microfluidic systems is still in its early stages of development. However, microfluidic devices can damage EVs due to shear stress and require macroscale samples [10].

## 5. Morphological Characterization of Extracellular Vesicles

Clinically relevant properties of membrane vesicles are size, morphology, biochemical composition, and cellular origin. The techniques recommended by ISEV for EVs analysis include electron microscopy (TEM) or atomic force microscopy (AFM) and a particle enumeration technique, such as nanoparticle-tracking analysis (NTA) or dynamic light scattering (DLS). TEM and cryo-TEM are the most practiced EV-imaging techniques [16, 30, 106]. Cryo-TEM preserves integrity of EVs and avoids artifacts generated by fixation [107]. However, AFM is suitable for size detection of EVs in their physiologic state [30, 106] and in morphology characterization by imaging.

Optical methods are able to obtain relevant properties of single vesicles exploiting the wavelength of light and the refractive index of particles in a suspending medium. DLS determines the relative size distribution of EVs isolated because it better performs the size determination of monodisperse samples, i.e., samples containing particles of a particular size. On the contrary, detection of the size distribution of polydisperse samples is less accurate because the larger vesicles scatter light more efficiently than smaller ones that become undetectable [106]. NTA exploits the dispersion of light collected by an optical microscope that detects the movements of a single particle by showing them through a video sequence. It performs well for MVs larger than 50 nm, but detection of smaller ones is not possible [106]. Both DLS and NTA are capable of determining size distribution of MVs within minutes, but no biochemical information is obtained.

Flow cytometry (FC) is mostly applied to identify EVs, to determine their number as well as their cell of origin by multiparametric scattered light and fluorescence measurements [19, 106, 108, 109]. Actually, FC uses latex beads of known size and with light-scattering properties similar to those of EVs to calibrate, in a standardized manner, scatter parameters (forward-scatter, FSC and side-scatter, SSC) and establish the related gates [2, 110, 111]. In particular, in any specific pathological condition, a FC measurement can be performed to investigate the relationship between the number of released EVs in a body fluid and the pathology of interest in order to use EVs as biomarkers [30]. Moreover, based on these considerations, Fluorescent-Activated Cell Sorting (FACS) could be used both for identifying, gating, counting [30, 95, 108, 111], and sorting pure EVs detectable in any type of sample and stemming from any type of cell, using specific fluorophore-conjugated antibodies [112]. As matter of fact, FACS is commonly used for the EV immunophenotyping, thanks of its ability to detect different antigens at the same time, therefore allowing the characterization of specific vesicular subsets [108, 110, 113]. Many studies reported that EVs counts correlated with various diseases and pathological conditions. For this reason, it is necessary to improve and standardize FC measurements for subsequent studies of their roles* in vivo *in order to use them for diagnostic and prognostic purposes [30, 110, 111, 113]. Controlled preanalytical and analytical conditions are critical to ensure reproducible quantification of EVs by FC in the context of clinical trials; therefore care should be taken in sample preparation, centrifugation conditions, and freezing methods and in all analytical tools that could modify the structure and quantification of EVs [108, 110, 114]. In conclusion, in agreement with other studies we believe that FC could be a powerful method for detecting, counting, gating, and probably sorting EVs, even if many of these characterization techniques may be performed in parallel to gain more detailed information about the purity of the isolated vesicular suspensions [106, 115].

## 6. Mass Spectrometry (MS)-Based Analysis of Extracellular Vesicle Proteins

### 6.1. MS-Based Proteomics Workflows

In the last decade, advances in high-throughput approaches allowed the development of integrated “omics” studies for evaluating the association of genetic and phenotypic variability with disease sensitivity and analgesic response [116]. For this reason, “omics” approaches are considered as a promising tool both for revealing molecular pathways and for identifying and quantifying different expressed molecules in many pathophysiological contexts, independently from the multiple trigger factors. Proteomics may be used for the discovery of disease biomarkers, potential drug targets and new cellular and biological mechanisms [116]. Nowadays, the characterization of proteome dramatically accelerated thanks to improvements in reverse phase chromatography coupled to MS, which allows identifying thousands of proteins in a few micrograms of material [117]. In particular, bottom-up proteomics approaches have been used as the main analytical strategy. For example, with* shotgun proteomics, *proteins are extracted from biological source, digested into peptides and directly analyzed by LC-MS/MS [6, 116]. Specifically, current studies have highlighted that Filter-Assisted Sample Preparation (FASP) has improved performance of biological MS data [16, 118]. Moreover the high resolution MS analyzers, i.e., hybrid mass spectrometers, such as quadrupole time-of-flight (Q-Tof) and ion-trap like Orbitraps, show major advantages like excellent mass accuracy, high resolving power and fast duty cycles, thus they have become the most used for analyzing complex samples, as biological fluids, cells lysate or EVs [119].

On the other hand, top-down proteomics allows sequencing intact proteins and their proteoforms without the need for enzymatic digestion. This approach can be applied for studying low molecular weight (<39 kDa) proteins; for example it was used to characterize specific and different proteoforms present in EVs isolated from murine myeloid-derived suppressor cells comparing their protein cargo to the parental cells [6].

There are two main approaches to make MS quantitative for studying EVs: the stable isotope-based (chemical labeling, metabolic labeling) and label-free workflows. MS-based label-free strategies are performed in four basic steps: (1) isolation and purification of intact EVs; (2) vesicles lysis, protein extraction and enzymatic digestion; (3) peptide separation and MS analysis; (4) data processing by bioinformatics software to get a protein list for their identification, quantification and statistical analysis [16, 120]. Quantitation is best based on the extraction of chromatographic peak area from raw data, then the peptide mass is estimated and the peak files are sent to a peptide database search engine, e.g., Andromeda [121]. Results are reported into a table of identified proteins versus their abundance per sample. This processing is often performed by different software (e.g., MaxQuant, which is a free platform) using raw data file obtained from LC-MS/MS analysis [122, 123]. An example is the work by Sun and colleagues [13] who performed a quantitative comparative proteomics analysis on salivary MVs and Exo of lung cancer patients* vs *healthy controls.

**Figure 3:**
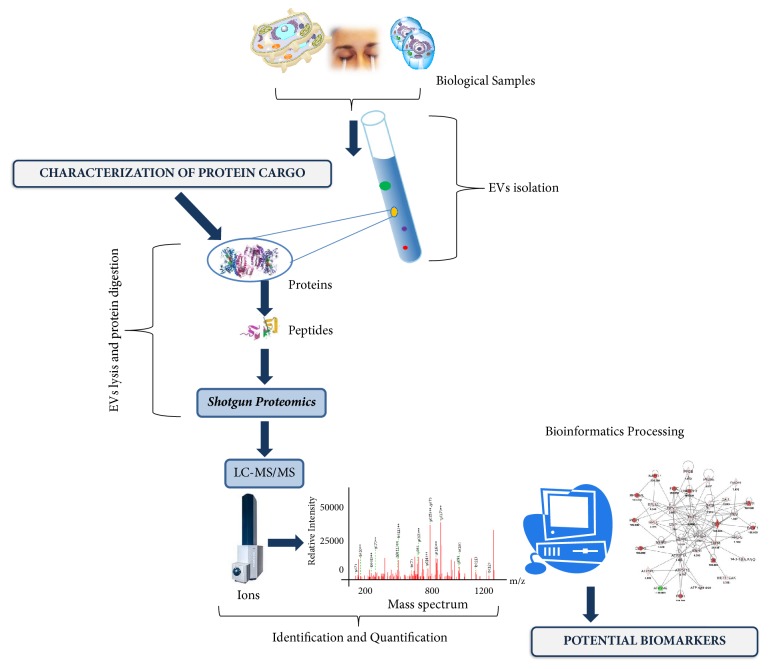
*An example of workflow for biomarker discovery process based on purification and proteomics characterization of EVs isolated from various biological samples*. After vesicles lysis, protein digestion is performed to separate the peptides that are analyzed through proteomics strategies. High resolution LC-MS instruments allow obtaining a protein list that can be identified and quantified by powerful bioinformatics software. Finally functional enrichment analysis identifies local networks and potential biomarkers. EVs, Extracellular Vesicles; LC-MS/MS, liquid chromatography coupled with online tandem mass spectrometry.

After that, during processing times, the MS/MS spectra are commonly searched using spectral libraries and specific bioinformatics software to understand multiple features of the identified proteins, such as posttranslational modifications (PTMs) and protein-protein interactions (PPIs). Whereas proteins do not act as single entity within a cell, but they build interaction networks which influence the phenotype, the PPI networks are crucial to understand the phenotype and the role of EVs in a complex biological system [6]. For this reason, in the last few years, MS-based proteomics methods have been developed to study PPIs becoming the approach of choice for large scale studies. Subcellular localization, biological pathways, biological functions, cellular components of the proteins can be easily analyzed using the annotations of the UniProt/SwissProt database [124, 125] thanks to enrichment analyses based on functional Gene Ontology (GO) approach, in order to build functional interaction networks between gene-gene relationships. Thus, many bioinformatics and robust software tools are available to process “omics” data for pathway analysis generating molecular networks. For example Ingenuity Pathway Analysis (IPA, Ingenuity® Systems, CA, USA) generates the biological pathways, associated with the proteins found with “omic” analysis, using computational algorithms for identifying local networks [116]. Many specific platforms have been developed for EVs research as EVpedia (evpedia.info) [126], ExoCarta (http://www.exocarta.org) [127], Vesiclepedia (http://www.microvesicles.org) [128] for providing a summary of proteins, lipids and RNA which have been identified in several EVs studies. The workflow in Figure 3 shows the main steps to be followed for the characterization of protein cargo of EVs isolated from various biological samples.

### 6.2. Protein Cargo of EVs

The recent emerging role of EVs in many pathophysiological processes calls for the precise characterization of their protein cargo. EVs content is not casual because these nanosized membrane vesicles are involved in cell-to-cell communication and transmit signals through the proteins, lipids, nucleic acids and sugars they transport. Indeed their proteome strongly influences their biological properties [6]. Protein components of EVs may be analyzed with different technologies, including but not limited to western blotting, FACS, immune-electron microscopy [17, 120]. Proteomics studies show that EVs cargo is dependent not only on the cell type of origin and physiological or pathological conditions, but also on the type of EVs Table 3 summarize the main EVs proteomics studies for biomarker discovery, highlighting the pathological condition, and the biofluid used, with the aim of schematize obtained results.

Furthermore proteins from different subcellular compartments are not equally represented in EVs. Actually, Raimondo et al. [129] found that cytoplasmic proteins are the most abundant in EVs isolated from biological fluids (47%) and cell culture media (43%), while membrane proteins represent 28% and 34% respectively. Nuclear and mitochondrial proteins are usually present in much lower amounts. Gene Ontology (GO) analysis of the top 100 proteins identified in exosomal preparations according to the ExoCarta database has showed the presence of effector components such as ADP Rybosilation Factors family (ARF) proteins involved in the vesicle biogenesis and intracellular trafficking and Miro and Ras family GTPases involved in vesicles biogenesis [6, 35].

Previous proteomics studies revealed “specific” Exo markers, such as tetraspanins (CD9, CD63, and CD81), binding protein Alix, Endosomal Sorting Complexes Required for Transport (ESCRTs) [6, 16, 17], even if these proteins have also been identified in both MVs and ABs [16, 28]. Because of confusing nomenclature, proteomics data from MVs and ABs are underrepresented in the scientific literature compared to the number of publications centered on Exo. In the light of MV biogenesis, their proteome reflects the cell of origin. Furthermore, as compared to Exo, MVs differ in their protein content because they are rich in proteins associated with microtubules, cortical activity and cytoskeleton networks [30, 35, 130]. Histones are sometimes found in MVs fractions and this could suggest that these fractions may contain some ABs (50-500 nm) since their size overlap with that of MVs (100-1000 nm) [130]. This might also indicate the presence of DNA in these vesicles, which could represent relevant information for the target cells. When Exo- and MVs-associated proteins were analyzed by the IPA platform, the most evidently emerging biological functions were: cellular movement, cell-to-cell signaling, tissue development, cancer and viral infections, leading the hypothesis that MVs may be released during apoptosis and pathophysiological processing more than Exo [30].

## 7. Conclusions and Future Directions

This review aimed to summarize the world around EVs and their isolation and characterization techniques, focusing in particular on the proteomic strategies available for EVs protein characterization (as reported in Table 3). High accuracy, sensitive, and robust bottom-up proteomic technologies have allowed improving our knowledge about EVs protein cargo. However, nowadays, characterization of specific vesicular subsets with current standardized isolation techniques is still unfeasible. In other words, the inconsistency of results reported so far in different works largely depends on preanalytical errors and technological issues related to EVs measurement and isolation, and above all on previous ambiguity in EVs definition (MVs* vs *Exo) [17].

Therefore, more studies are needed to better evaluate the applicability of EVs as biomarkers in translational diagnostic. Furthermore, their ability to transport molecules and to target specific cells raises intriguing scenarios about their development as therapeutics and in drug discovery [15, 35]. Proteomics strategies combined with other “omics” approaches, such as metabolomics, genomics and transcriptomics, could be used for rapid quantitative analyses of EVs molecular panels, signaling pathways, and pharmacokinetics. Moreover one of the main goals in the field is to improve the sequence coverage of the EVs proteome in order to better characterize their molecular cargo by identifying PTMs, mutations and specific proteoforms in particular pathological states.

In conclusion, this review highlighted that cancer cells are able to generate EVs* in vivo *that functionally cooperate to transform phenotype of recipient cells by reprogramming them and thus conferring some of the characteristics of cancer cells (i.e., fibroblasts and epithelial cells) [131, 132]. These vesicles are potential mediators of many and not completely understood tumor-related phenomena such as intravasation and extravasation, angiogenesis, and preparation of distant sites before implantation of metastatic cells, response to therapy, and immunomodulation [55]. Proteomics approaches will be useful in elucidate such a phenomena; however future progress in the optimization and standardization of EVs enrichment is needed. Better purification methods will increase functional and phenotypic characterization that, in turn, will allow the use of EVs in the clinical practice for diagnosis and therapy, opening the way to an improved patients' stratification in many multifactorial diseases as cancer [56].

## Figures and Tables

**Table 1:** Overview of the main characteristics of different types of extracellular membranous vesicles.

	Exo	MVs	ABs	LOs
Size (diameter)	30-100 nm [19]	100-1000 nm [3, 19, 30]	50-500 nm [32]	1-10 *μ*m [9, 33]
Flotation density	1.10-1.21 g/mL	NA	1.16-1.28 g/mL	NA
Morphology	*“cup-shaped”*	Various shapes	Heterogeneous [3, 32]	Large size - Various shapes
Lipid composition	Cholesterol, ceramide, sphingomyelin, low phosphatidylserine exposure, lipid rafts	High phosphatidylserine exposure, cholesterol [20, 28, 34]	High phosphatidylserine exposure	High phosphatidylserine exposure, cholesterol
Protein markers	Alix, CD63, CD9, CD81 [17, 35]	Selectins, integrins, CD40, MMP	Histones [32]	ARF6, CK18, GAPDH, MMP, oncogenic proteins complexes [31, 33, 36]
Site of origin	MVEs [1] or MVBs [17]	Plasma membrane	-	Plasma membrane
Mode of extracellular release	Exocytosis of MVEs [23]	Budding/blebbing of the plasma membrane [28]	Cell shrinkage and death	Budding from the plasma membrane [9, 33]
Composition	Proteins, miRNA, mRNA	Proteins, miRNA, mRNA [8]	Proteins, DNA [32], miRNA, RNA [8]	Proteins, miRNA, mRNA, DNA [36, 37]

MVs, microvesicles; ABs, apoptotic blebs; LOs, large oncosomes; MVEs, multivesicular endosomes; MVBs, multivesicular bodies; MMP, metalloproteinases; NA, not known.

**Table 2:** Established methods of EVs isolation and purification.

Method	Advantages	Disadvantages
Differential ultracentrifugation	Commonly used method allowing comparison between studies [17, 19, 38]	Slow and laborious technique Includes contaminants without additional steps EVs may aggregate [6, 10, 39] Pellet can be difficult to resuspend [10]
Density gradient ultracentrifugation	Commonly used method allowing comparison between studies Products of higher purity than differential ultracentrifugation [10, 19, 40]	Slow and laborious technique [10, 41] Some media, for example, sucrose, may interfere with EVs function [6]
Ultrafiltration	Concentrates large volumes Cleans up the samples before other analyses [13, 40, 42]	Potential losses under high pressure and unspecific membrane adsorption [6]
Immunoaffinity capture	Highly pure product Rapid Used for immunophenotyping EV s [10, 20, 40]	Costly Low yield Need to remove EVs from antibodies which may mask what required for target selection or effect [10, 16]
Precipitation or “salting out”	Does not require specialized equipment Rapid [10, 40]	Relatively impure products Excess of salt and polymer can interfere with subsequent analyses [6, 16]
Size exclusion chromatography	Good separation [10, 16, 40]	Need to concentrate the samples [11]
Microfluidics techniques	Rapid Ideal for industrial manufacture [10, 40]	Shear stress can damage EVs structure [10]

**Table 3:** Summary of the most significant study designs used to analyze the EV proteome, including potential biomarkers identified.

Reference	Biofluid	Pathological condition	Isolation technique	EV subtype	MS-based strategy	Candidate biomarkers
Sun *et al. *[43]	Saliva and serum	Lung Cancer	PureEXO® isolation kit (101Bio)	Exo	ESI-QTOF MS (MaXis Impact) LC-MS/MS (Q Exactive)	Vimentin, Phospholipid transfer protein, Annexin, Zinc-alpha-2-glycoprotein, Lactoperoxidase, Proteasome subunit alpha, Grancalcin, Cysteine-rich secretory protein 3, Calpain small subunit 1, Histone H3
Sun *et al.* [13]	Saliva	Lung Cancer	Ultracentrifugation	Exo and MVs	LC-MS/MS (Q Exactive)	Ras GTPase-activating-like protein, BPI fold-containing family A member 1, Cornulin, Mucin-5B
Raimondo *et al.* [44]	Urine	Renal Cell Carcinoma	Iodixanol density centrifugation	Exo	LC-ESI-MS/MS (MaXis hybrid UHR-QTOF)	Matrix metalloproteinase-9, Ceruloplasmin, Podocalyxin, Dickkopf related protein 4, Carbonic Anhydrase IX, Aquaporin-1, Extracellular Matrix Metalloproteinase Inducer, Neprilysin, Dipeptidase 1, Syntenin-1
Chen *et al.* [45]	Serum	Colorectal Cancer	Ultracentrifugation	Exo	HPLC-MS/MS (Orbitrap Fusion)	Fibronectin-1, Annexin-A1, Metalloproteinase-9, Galectin-3 binding protein, Insulin-like growth factor
Aqrawi *et al.* [11]	Saliva and tear	Sjögren's Syndrome	Size-exclusion chromatography	Exo and MVs	LC-MS/MS (Q Exactive)	Lymphocyte-specific protein 1, Adipocyte plasma membrane-associated protein, Copine-1, Thioredoxin-dependent peroxidase reductase
Choi *et al.* [46]	Ascites	Colorectal Cancer	Iodixanol and sucrose density centrifugation	MVs	LC-ESI-MS/MS (LTQ)	Carcinoembryonic antigen cell adhesion molecule 5, Adhesion G-protein-coupled receptor E5, Galectin-3, Epithelial cell adhesion molecule, Aminopeptidase N, Trophoblast glycoprotein
Kittivorapart *et al.* [47]	Plasma	HbE/*β*- thalassaemia	Ultracentrifugation	Non specified	HPLC-MS/MS (Orbitrap Fusion)	Alpha hemoglobin–stabilizing protein, Catalase, Superoxide dismutase, Hemopexin, Haptoglobin
Shiromizu *et al. *[48]	Serum	Colorectal Cancer	Sucrose density centrifugation	Not specified	LC-MS/MS (Q Exactive)	Annexin-A3, A4, A5 and A11, Tenascin-N, Mucin-5B, Matrix metalloproteinase-9, Transferrin receptor protein 1
Sequeiros *et al. *[49]	Urine	Prostate Cancer	Ultracentrifugation	Not specified	SRM-MS (5500 Q-Trap)	Adseverin, Transglutaminase-4
Arbelaiz *et al.* [50]	Serum	Cholangiocarcinoma (CCA) Primary Sclerosing Cholangitis (PSC) Hepatocellular carcinoma (HCC)	Ultracentrifugation	Not specified	UPLC-MS/MS (LTQ Orbitrap XL and Synapt G2-Si)	CCA: Aminopeptidase N, Pantetheinase, Polymeric immunoglobulin receptor PSC: Ficolin-1, Neprilysin, Fibrinogen gamma chain, Alpha-1-acid glycoprotein 1, S100A8 proteins HCC: Galectin-3-binding protein, Polymeric immunoglobulin receptor
Yang * et al. *[51]	Seminal fluid	Prostate Cancer	Sucrose density centrifugation	Exo	HPLC-MS/MS (LTQ Orbitrap Elite)	Semenogelin-1, Phosphoglycerate dehydrogenase, Galectin-3-binding protein, Actin, Glyceraldehyde-3-phosphate dehydrogenase
Barnabas *et al.* [52]	Uterine Liquid Biopsy	Ovarian Cancer	Ultracentrifugation	MVs	LC-MS/MS (Q Exactive)	Calcium-activated chloride channel regulator 4, Involucrin, Protein S100-A14, Protein S100-A2, Serpin B5
Turay *et al.* [53]	Plasma	Prostate Cancer	Exoquick™ Isolation Kit	Exo	HPLC-MS/MS (LCQ Deca XP)	Iroquois homeobox protein 5, Mitochondrial tumor suppressor 1 isoform 4, Trinucleotide repeat containing 6B isoform 3

ESI, electrospray ionization; QTOF, quadrupole time-of-flight; LTQ, linear trap quadrupole; SRM, selected reaction monitoring.

## References

[1] van der Pol E., Böing A. N., Harrison P., Sturk A., Nieuwland R. (2012). Classification, functions, and clinical relevance of extracellular vesicles. *Pharmacological Reviews*.

[2] Hargett L. A., Bauer N. N. (2013). On the origin of microparticles: from ‘platelet dust’ to mediators of intercellular communication. *Pulmonary Circulation*.

[3] Wolf P. (1967). The nature and significance of platelet products in human plasma.. *British Journal of Haematology*.

[4] Shah R., Patel T., Freedman J. E. (2018). Circulating extracellular vesicles in human disease. *The New England Journal of Medicine*.

[5] Lötvall J., Hill A. F., Hochberg F. (2014). Minimal experimental requirements for definition of extracellular vesicles and their functions: a position statement from the International Society for Extracellular Vesicles. *Journal of Extracellular Vesicles (JEV)*.

[6] Rosa-Fernandes L., Rocha V. B., Carregari V. C., Urbani A., Palmisano G. (2017). A perspective on extracellular vesicles proteomics. *Frontiers in Chemistry*.

[7] Montoro-García S., Shantsila E., Marín F., Blann A., Lip G. Y. H. (2011). Circulating microparticles: New insights into the biochemical basis of microparticle release and activity. *Basic Research in Cardiology*.

[8] Ratajczak J., Wysoczynski M., Hayek F., Janowska-Wieczorek A., Ratajczak M. Z. (2006). Membrane-derived microvesicles: important and underappreciated mediators of cell-to-cell communication. *Leukemia*.

[9] Minciacchi V. R., Freeman M. R., Di Vizio D. (2015). Extracellular vesicles in cancer: exosomes, microvesicles and the emerging role of large oncosomes. *Seminars in Cell & Developmental Biology*.

[10] Kang H., Kim J., Park J. (2017). Methods to isolate extracellular vesicles for diagnosis. *Micro and Nano Systems Letters*.

[11] Aqrawi L. A., Galtung H. K., Vestad B. (2017). Identification of potential saliva and tear biomarkers in primary Sjögren’s syndrome, utilising the extraction of extracellular vesicles and proteomics analysis. *Arthritis Research & Therapy*.

[12] Yuana Y., Sturk A., Nieuwland R. (2013). Extracellular vesicles in physiological and pathological conditions. *Blood Reviews*.

[13] Sun Y., Huo C., Qiao Z. (2018). Comparative proteomic analysis of exosomes and microvesicles in human saliva for lung cancer. *Journal of Proteome Research*.

[14] Carandini T., Colombo F., Finardi A. (2015). Microvesicles: what is the role in multiple sclerosis?. *Frontiers in Neurology*.

[15] Reiner A. T., Witwer K. W., van Balkom B. W. (2017). Concise review: developing best-practice models for the therapeutic use of extracellular vesicles. *Stem Cells Translational Medicine*.

[16] Sódar B. W., Kovács Á., Visnovitz T. (2017). Best practice of identification and proteomic analysis of extracellular vesicles in human health and disease. *Expert Review of Proteomics*.

[17] Mathivanan S., Ji H., Simpson R. J. (2010). Exosomes: extracellular organelles important in intercellular communication. *Journal of Proteomics*.

[18] Gould S. J., Raposo G. (2013). As we wait: Coping with an imperfect nomenclature for extracellular vesicles. *Journal of Extracellular Vesicles (JEV)*.

[19] Barrachina M. N., Calderón-Cruz B., Fernandez-Rocca L., García Á. (2019). Application of extracellular vesicles proteomics to cardiovascular disease: guidelines, data analysis, and future perspectives. *Proteomics*.

[20] Akers J. C., Gonda D., Kim R., Carter B. S., Chen C. C. (2013). Biogenesis of extracellular vesicles (EV): exosomes, microvesicles, retrovirus-like vesicles, and apoptotic bodies. *Journal of Neuro-Oncology*.

[21] Johnstone R. M., Adam M., Hammond J. R., Orr L., Turbide C. (1987). Vesicle formation during reticulocyte maturation. Association of plasma membrane activities with released vesicles (exosomes). *The Journal of Biological Chemistry*.

[22] Pan B.-T., Teng K., Wu C., Adam M., Johnstone R. M. (1985). Electron microscopic evidence for externalization of the transferrin receptor in vesicular form in sheep reticulocytes. *The Journal of Cell Biology*.

[23] Raposo G., Stoorvogel W. (2013). Extracellular vesicles: exosomes, microvesicles, and friends. *The Journal of Cell Biology*.

[24] Baietti M. F., Zhang Z., Mortier E. (2012). Syndecan-syntenin-ALIX regulates the biogenesis of exosomes. *Nature Cell Biology*.

[25] Raiborg C., Stenmark H. (2009). The ESCRT machinery in endosomal sorting of ubiquitylated membrane proteins. *Nature*.

[26] Juan T., Fürthauer M. (2018). Biogenesis and function of ESCRT-dependent extracellular vesicles. *Seminars in Cell & Developmental Biology*.

[27] Abrahams V. M., Straszewski S. L., Kamsteeg M. (2003). Epithelial ovarian cancer cells secrete functional Fas ligand. *Cancer Research*.

[28] Van Niel G., D'Angelo G., Raposo G. (2018). Shedding light on the cell biology of extracellular vesicles. *Nature Reviews Molecular Cell Biology*.

[29] Camussi G., Deregibus M. C., Bruno S., Cantaluppi V., Biancone L. (2010). Exosomes/microvesicles as a mechanism of cell-to-cell communication. *Kidney International*.

[30] György B., Szabó T. G., Pásztói M. (2011). Membrane vesicles, current state-of-the-art: emerging role of extracellular vesicles. *Cellular and Molecular Life Sciences*.

[31] Ciardiello C., Cavallini L., Spinelli C. (2016). Focus on extracellular vesicles: New frontiers of cell-to-cell communication in cancer. *International Journal of Molecular Sciences*.

[32] Kerr J. F., Wyllie A. H., Currie A. R. (1972). Apoptosis: a basic biological phenomenon with wide-ranging implications in tissue kinetics. *British Journal of Cancer*.

[33] Minciacchi V. R., You S., Spinelli C. (2015). Large oncosomes contain distinct protein cargo and represent a separate functional class of tumor-derived extracellular vesicles. *Oncotarget*.

[34] Al-Nedawi K., Meehan B., Rak J. (2009). Microvesicles: messengers and mediators of tumor progression. *Cell Cycle*.

[35] Greening D. W., Simpson R. J. (2018). Understanding extracellular vesicle diversity – current status. *Expert Review of Proteomics*.

[36] Becker A., Thakur B. K., Weiss J. M., Kim H. S., Peinado H., Lyden D. (2016). Extracellular Vesicles in cancer: cell-to-cell mediators of metastasis. *Cancer Cell*.

[37] Vagner T., Spinelli C., Minciacchi V. R. (2018). Large extracellular vesicles carry most of the tumour DNA circulating in prostate cancer patient plasma. *Journal of Extracellular Vesicles (JEV)*.

[38] Raposo G., Nijman H. W., Stoorvogel W. (1996). B lymphocytes secrete antigen-presenting vesicles. *The Journal of Experimental Medicine*.

[39] Linares R., Tan S., Gounou C., Arraud N., Brisson A. R. (2015). High-speed centrifugation induces aggregation of extracellular vesicles. *Journal of Extracellular Vesicles (JEV)*.

[40] Li P., Kaslan M., Lee S. H., Yao J., Gao Z. (2017). Progress in exosome isolation techniques. *Theranostics*.

[41] Cvjetkovic A., Lötvall J., Lässer C. (2014). The influence of rotor type and centrifugation time on the yield and purity of extracellular vesicles. *Journal of Extracellular Vesicles (JEV)*.

[42] Lane R. E., Korbie D., Trau M. (2017). Purification protocols for extracellular vesicles. *Methods in Molecular Biology*.

[43] Sun Y., Liu S., Qiao Z. (2017). Systematic comparison of exosomal proteomes from human saliva and serum for the detection of lung cancer. *Analytica Chimica Acta*.

[44] Raimondo F., Morosi L., Corbetta S. (2013). Differential protein profiling of renal cell carcinoma urinary exosomes. *Molecular BioSystems*.

[45] Chen Y., Xie Y., Xu L. (2017). Protein content and functional characteristics of serum-purified exosomes from patients with colorectal cancer revealed by quantitative proteomics. *International Journal of Cancer*.

[46] Choi D.-S., Park J. O., Jang S. C. (2011). Proteomic analysis of microvesicles derived from human colorectal cancer ascites. *Proteomics*.

[47] Kittivorapart J., Crew V. K., Wilson M. C., Heesom K. J., Siritanaratkul N., Toye A. M. (2018). Quantitative proteomics of plasma vesicles identify novel biomarkers for hemoglobin E/*β*-thalassemic patients. *Blood Advances*.

[48] Shiromizu T., Kume H., Ishida M. (2017). Quantitation of putative colorectal cancer biomarker candidates in serum extracellular vesicles by targeted proteomics. *Scientific Reports*.

[49] Sequeiros T., Rigau M., Chiva C. (2017). Targeted proteomics in urinary extracellular vesicles identifies biomarkers for diagnosis and prognosis of prostate cancer. *Oncotarget *.

[50] Arbelaiz A., Azkargorta M., Krawczyk M. (2017). Serum extracellular vesicles contain protein biomarkers for primary sclerosing cholangitis and cholangiocarcinoma. *Hepatology*.

[51] Yang C., Guo W.-B., Zhang W.-S. (2017). Comprehensive proteomics analysis of exosomes derived from human seminal plasma. *Andrology*.

[52] Barnabas G. D., Bahar-Shany K., Sapoznik S. (2019). Microvesicle proteomic profiling of uterine liquid biopsy for ovarian cancer early detection. *Molecular & Cellular Proteomics*.

[53] Turay D., Khan S., Diaz Osterman C. J. (2016). Proteomic profiling of serum-derived exosomes from ethnically diverse prostate cancer patients. *Cancer Investigation*.

[54] Minciacchi V. R., Spinelli C., Reis-Sobreiro M. (2017). MYC mediates large oncosome-induced fibroblast reprogramming in prostate cancer. *Cancer Research*.

[55] Di Vizio D., Morello M., Dudley A. C. (2012). Large oncosomes in human prostate cancer tissues and in the circulation of mice with metastatic disease. *The American Journal of Pathology*.

[56] Li I., Nabet B. Y. (2019). Exosomes in the tumor microenvironment as mediators of cancer therapy resistance. *Molecular Cancer*.

[57] Koga K., Matsumoto K, Akiyoshi T. (2005). Purification, characterization and biological significance of tumor-derived exosomes. *Anticancer Research*.

[58] Selmaj I., Mycko M. P., Raine C. S., Selmaj K. W. (2017). The role of exosomes in CNS inflammation and their involvement in multiple sclerosis. *Journal of Neuroimmunology*.

[59] Lee J., McKinney K. Q., Pavlopoulos A. J. (2016). Exosomal proteome analysis of cerebrospinal fluid detects biosignatures of neuromyelitis optica and multiple sclerosis. *Clinica Chimica Acta*.

[60] Kanninen K. M., Bister N., Koistinaho J., Malm T. (2016). Exosomes as new diagnostic tools in CNS diseases. *Biochimica et Biophysica Acta (BBA) - Molecular Basis of Disease*.

[61] Cai Z., Yang F., Yu L. (2012). Activated T cell exosomes promote tumor invasion via Fas signaling pathway. *The Journal of Immunology*.

[62] Abusamra A. J., Zhong Z., Zheng X. (2005). Tumor exosomes expressing Fas ligand mediate CD8+ T-cell apoptosis. *Blood Cells, Molecules, and Diseases*.

[63] Kim J. W., Wieckowski E., Taylor D. D. (2005). Fas ligand-positive membranous vesicles isolated from sera of patients with oral cancer induce apoptosis of activated T lymphocytes. *Clinical Cancer Research*.

[64] Friend C., Marovitz W., Henie G. (1978). Observations on cell lines derived from a patient with Hodgkin's disease. *Cancer Research*.

[65] Bandu R., Oh J. W., Kim K. P. (2019). Mass spectrometry-based proteome profiling of extracellular vesicles and their roles in cancer biology. *Experimental & Molecular Medicine*.

[66] Al-Nedawi K., Meehan B., Micallef J. (2008). Intercellular transfer of the oncogenic receptor EGFRvIII by microvesicles derived from tumour cells. *Nature Cell Biology*.

[67] Zhao H., Achreja A., Iessi E. (2018). The key role of extracellular vesicles in the metastatic process. *Biochimica et Biophysica Acta (BBA) - Reviews on Cancer*.

[68] Webber J. P., Spary L. K., Sanders A. J. (2015). Differentiation of tumour-promoting stromal myofibroblasts by cancer exosomes. *Oncogene*.

[69] Antonyak M. A., Li B., Boroughs L. K. (2011). Cancer cell-derived microvesicles induce transformation by transferring tissue transglutaminase and fibronectin to recipient cells. *Proceedings of the National Acadamy of Sciences of the United States of America*.

[70] Hakulinen J., Sankkila L., Sugiyama N., Lehti K., Keski-Oja J. (2008). Secretion of active membrane type 1 matrix metalloproteinase (MMP-14) into extracellular space in microvesicular exosomes. *Journal of Cellular Biochemistry*.

[71] De Rubis G., Rajeev Krishnan S., Bebawy M. (2019). Liquid Biopsies in Cancer Diagnosis, Monitoring, and Prognosis. *Trends in Pharmacological Sciences*.

[72] Chen G., Huang A. C., Guo W. (2018). Exosomal PD-L1 contributes to immunosuppression and is associated with anti-PD-1 response. *Nature*.

[73] Batista I., Melo S. (2019). Exosomes and the Future of Immunotherapy in Pancreatic Cancer. *International Journal of Molecular Sciences*.

[74] Maybruck B. T., Pfannenstiel L. W., Diaz-Montero M., Gastman B. R. (2017). Tumor-derived exosomes induce CD8+ T cell suppressors. *Journal for ImmunoTherapy of Cancer*.

[75] Raimondo S., Giavaresi G., Lorico A., Alessandro R. (2019). Extracellular Vesicles as Biological Shuttles for Targeted Therapies. *International Journal of Molecular Sciences*.

[76] Usman W. M., Pham T. C., Kwok Y. Y. (2018). Efficient RNA drug delivery using red blood cell extracellular vesicles. *Nature Communications*.

[77] Melo S. A., Luecke L. B., Kahlert C. (2015). Glypican-1 identifies cancer exosomes and detects early pancreatic cancer. *Nature*.

[78] Wang L., Duan W., Yan S., Xie Y., Wang C. (2019). Circulating long non-coding RNA colon cancer-associated transcript 2 protected by exosome as a potential biomarker for colorectal cancer. *Biomedicine & Pharmacotherapy*.

[79] Dai S., Wan T., Wang B. (2005). More efficient induction of HLA-A∗0201-restricted and carcinoembryonic antigen (CEA) - Specific CTL response by immunization with exosomes prepared from heat-stressed CEA-positive tumor cells. *Clinical Cancer Research*.

[80] Tauro B. J., Greening D. W., Mathias R. A., Mathivanan S., Ji H., Simpson R. J. (2013). Two distinct populations of exosomes are released from LIM1863 colon carcinoma cell-derived organoids. *Molecular & Cellular Proteomics*.

[81] Mousavi S., Moallem R., Hassanian S. M. (2019). Tumor-derived exosomes: Potential biomarkers and therapeutic target in the treatment of colorectal cancer. *Journal of Cellular Physiology*.

[82] Toiyama Y., Takahashi M., Hur K. (2013). Serum miR-21 as a diagnostic and prognostic biomarker in colorectal cancer. *Journal of the National Cancer Institute*.

[83] Tanaka Y., Kamohara H., Kinoshita K. (2013). Clinical impact of serum exosomal microRNA-21 as a clinical biomarker in human esophageal squamous cell carcinoma. *Cancer*.

[84] Fu M., Gu J., Jiang P., Qian H., Xu W., Zhang X. (2019). Exosomes in gastric cancer: roles, mechanisms, and applications. *Molecular Cancer*.

[85] Qu J.-L., Qu X.-J., Zhao M.-F. (2009). Gastric cancer exosomes promote tumour cell proliferation through PI3K/Akt and MAPK/ERK activation. *Digestive and Liver Disease*.

[86] Miki Y., Yashiro M., Okuno T. (2018). Clinico-pathological significance of exosome marker CD63 expression on cancer cells and stromal cells in gastric cancer. *PLoS ONE*.

[87] Rupp A.-K., Rupp C., Keller S. (2011). Loss of EpCAM expression in breast cancer derived serum exosomes: role of proteolytic cleavage. *Gynecologic Oncology*.

[88] Meng Y., Sun J., Wang X. (2019). Exosomes: a promising avenue for the diagnosis of breast cancer. *Technology in Cancer Research & Treatment*.

[89] Moon P.-G., Lee J.-E., Cho Y.-E. (2016). Fibronectin on circulating extracellular vesicles as a liquid biopsy to detect breast cancer. *Oncotarget *.

[90] Moon P.-G., Lee J.-E., Cho Y.-E. (2016). Identification of developmental endothelial locus-1 on circulating extracellular vesicles as a novel biomarker for early breast cancer detection. *Clinical Cancer Research*.

[91] Muller L., Hong C.-S., Stolz D. B., Watkins S. C., Whiteside T. L. (2014). Isolation of biologically-active exosomes from human plasma. *Journal of Immunological Methods*.

[92] Ruhen O., Meehan K. (2019). Tumor-derived extracellular vesicles as a novel source of protein biomarkers for cancer diagnosis and monitoring. *Proteomics*.

[93] Li Y., Zhang Y., Qiu F., Qiu Z. (2011). Proteomic identification of exosomal LRG1: A potential urinary biomarker for detecting NSCLC. *Electrophoresis*.

[94] Welton J. L., Loveless S., Stone T., von Ruhland C., Robertson N. P., Clayton A. (2017). Cerebrospinal fluid extracellular vesicle enrichment for protein biomarker discovery in neurological disease; multiple sclerosis. *Journal of Extracellular Vesicles (JEV)*.

[95] Lener T., Gimona M., Aigner L. (2015). Applying extracellular vesicles based therapeutics in clinical trials - an ISEV position paper. *Journal of Extracellular Vesicles*.

[96] Mathivanan S., Lim J. W. E., Tauro B. J., Ji H., Moritz R. L., Simpson R. J. (2010). Proteomics analysis of A33 immunoaffinity-purified exosomes released from the human colon tumor cell line LIM1215 reveals a tissue-specific protein signature. *Molecular & Cellular Proteomics*.

[97] Brady J. V., Troyer R. M., Ramsey S. A. (2018). A preliminary proteomic investigation of circulating exosomes and discovery of biomarkers associated with the progression of osteosarcoma in a clinical model of spontaneous disease. *Translational Oncology*.

[98] Rider M. A., Hurwitz S. N., Meckes Jr. D. G. (2016). ExtraPEG: A polyethylene glycol-based method for enrichment of extracellular vesicles. *Scientific Reports*.

[99] Markowska A., Pendergrast R. S., Pendergrast J. S., Pendergrast P. S. (2017). A novel method for the isolation of extracellular vesicles and RNA from urine. *Journal of Circulating Biomarkers*.

[100] Niu Z., Pang R. T., Liu W. (2017). Polymer-based precipitation preserves biological activities of extracellular vesicles from an endometrial cell line. *PLoS ONE*.

[101] Gallart-Palau X., Serra A., Sze S. K. (2016). Enrichment of extracellular vesicles from tissues of the central nervous system by PROSPR. *Molecular Neurodegeneration*.

[102] Gallart-Palau X., Serra A., Wong A. S. W. (2015). Extracellular vesicles are rapidly purified from human plasma by PRotein Organic Solvent PRecipitation (PROSPR). *Scientific Reports*.

[103] Rood I. M., Deegens J. K. J., Merchant M. L. (2010). Comparison of three methods for isolation of urinary microvesicles to identify biomarkers of nephrotic syndrome. *Kidney International*.

[104] Kanwar S. S., Dunlay C. J., Simeone D. M., Nagrath S. (2014). Microfluidic device (ExoChip) for on-chip isolation, quantification and characterization of circulating exosomes. *Lab on a Chip *.

[105] Wang Z., Wu H.-J., Fine D. (2013). Ciliated micropillars for the microfluidic-based isolation of nanoscale lipid vesicles. *Lab on a Chip *.

[106] Van Der Pol E., Hoekstra A. G., Sturk A., Otto C., Van Leeuwen T. G., Nieuwland R. (2010). Optical and non-optical methods for detection and characterization of microparticles and exosomes. *Journal of Thrombosis and Haemostasis*.

[107] Merchant M. L., Rood I. M., Deegens J. K. J., Klein J. B. (2017). Isolation and characterization of urinary extracellular vesicles: Implications for biomarker discovery. *Nature Reviews Nephrology*.

[108] Gamonet C., Mourey G., Aupet S. (2017). How to quantify microparticles in RBCs? A validated flow cytometry method allows the detection of an increase in microparticles during storage. *Transfusion*.

[109] de Rond L., Coumans F. A., Nieuwland R., van Leeuwen T. G., van der Pol E. (2018). Deriving extracellular vesicle size from scatter intensities measured by flow cytometry. *Current Protocols in Cytometry*.

[110] Santilli F., Marchisio M., Lanuti P., Boccatonda A., Miscia S., Davl G. (2016). Microparticles as new markers of cardiovascular risk in diabetes and beyond. *Thrombosis and Haemostasis*.

[111] Pieragostino D., Cicalini I., Lanuti P. (2018). Enhanced release of acid sphingomyelinase-enriched exosomes generates a lipidomics signature in CSF of Multiple Sclerosis patients. *Scientific Reports*.

[112] Varga Z., van der Pol E., Pálmai M. (2018). Hollow organosilica beads as reference particles for optical detection of extracellular vesicles. *Journal of Thrombosis and Haemostasis*.

[113] Morales-Kastresana A., Telford B., Musich T. A. (2017). Labeling extracellular vesicles for nanoscale flow cytometry. *Scientific Reports*.

[114] Mullier F., Bailly N., Chatelain C., Chatelain B., Dogné J.-M. (2013). Pre-analytical issues in the measurement of circulating microparticles: Current recommendations and pending questions. *Journal of Thrombosis and Haemostasis*.

[115] Gyorgy B., Módos K., Pállinger E. (2011). Detection and isolation of cell-derived microparticles are compromised by protein complexes resulting from shared biophysical parameters. *Blood*.

[116] Del Boccio P., Rossi C., di Ioia M., Cicalini I., Sacchetta P., Pieragostino D. (2016). Integration of metabolomics and proteomics in multiple sclerosis: From biomarkers discovery to personalized medicine. *PROTEOMICS - Clinical Applications*.

[117] Di Palma S., Stange D., Van De Wetering M., Clevers H., Heck A. J. R., Mohammed S. (2011). Highly sensitive proteome analysis of FACS-sorted adult colon stem cells. *Journal of Proteome Research*.

[118] Erde J., Loo R. R. O., Loo J. A. (2014). Enhanced FASP (eFASP) to increase proteome coverage and sample recovery for quantitative proteomic experiments. *Journal of Proteome Research*.

[119] Whitham M., Febbraio M. A. (2019). Redefining tissue crosstalk via shotgun proteomic analyses of plasma extracellular vesicles. *Proteomics*.

[120] Pocsfalvi G., Stanly C., Vilasi A. (2016). Mass spectrometry of extracellular vesicles. *Mass Spectrometry Reviews*.

[121] Cox J., Neuhauser N., Michalski A., Scheltema R. A., Olsen J. V., Mann M. (2011). Andromeda: a peptide search engine integrated into the MaxQuant environment. *Journal of Proteome Research*.

[122] Cox J., Mann M. (2008). Maxquant enables high peptide identification rates, individualized ppb-range mass accuracies and proteome-wide protein quantification. *Nature Biotechnology*.

[123] Tyanova S., Temu T., Cox J. (2016). The MaxQuant computational platform for mass spectrometry-based shotgun proteomics. *Nature Protocols*.

[124] O'Donovan C., Martin M. J., Gattiker A., Gasteiger E., Bairoch A., Apweiler R. (2002). High-quality protein knowledge resource: SWISS-PROT and TrEMBL.. *Briefings in Bioinformatics*.

[125] The UniProt C. (2017). Uniprot: the universal protein knowledgebase. *Nucleic Acids Research*.

[126] Kim D.-K., Lee J., Kim S. R. (2015). EVpedia: a community web portal for extracellular vesicles research. *Bioinformatics*.

[127] Mathivanan S., Fahner C. J., Reid G. E., Simpson R. J. (2012). Exocarta 2012: database of exosomal proteins, RNA and lipids. *Nucleic Acids Research*.

[128] Kalra H., Simpson R. J., Ji H. (2012). Vesiclepedia: a compendium for extracellular vesicles with continuous community annotatio. *PLoS Biology*.

[129] Raimondo F., Morosi L., Chinello C., Magni F., Pitto M. (2011). Advances in membranous vesicle and exosome proteomics improving biological understanding and biomarker discovery. *Proteomics*.

[130] Korenevskii A. V., Milyutina Y. P., Zhdanova A. A., Pyatygina K. M., Sokolov D. I., Sel’kov S. A. (2018). Mass-spectrometric analysis of proteome of microvesicles produced by NK-92 natural killer cells. *Bulletin of Experimental Biology and Medicine*.

[131] Nogués L., Benito-Martin A., Hergueta-Redondo M., Peinado H. (2018). The influence of tumour-derived extracellular vesicles on local and distal metastatic dissemination. *Molecular Aspects of Medicine*.

[132] Zomer A., Maynard C., Verweij F. J. (2015). In vivo imaging reveals extracellular vesicle-mediated phenocopying of metastatic behavior. *Cell*.

